# On the Origin of ATP Synthesis in Cancer

**DOI:** 10.1016/j.isci.2020.101761

**Published:** 2020-11-02

**Authors:** Thomas N. Seyfried, Gabriel Arismendi-Morillo, Purna Mukherjee, Christos Chinopoulos

**Affiliations:** 1 Biology Department, Boston College, 140 Commonwealth Avenue, Chestnut Hill, MA 02467, USA; 2 Electron Microscopy Laboratory, Biological Researches Institute, Faculty of Medicine, University of Zulia, Maracaibo, Venezuela; 3 Department of Medical Biochemistry, Semmelweis University, Budapest, 1094, Hungary

**Keywords:** Biological Sciences, Biochemistry, Cell Biology, Cancer Systems Biology

## Abstract

ATP is required for mammalian cells to remain viable and to perform genetically programmed functions. Maintenance of the ΔG′_ATP_ hydrolysis of −56 kJ/mole is the endpoint of both genetic and metabolic processes required for life. Various anomalies in mitochondrial structure and function prevent maximal ATP synthesis through OxPhos in cancer cells. Little ATP synthesis would occur through glycolysis in cancer cells that express the dimeric form of pyruvate kinase M2. Mitochondrial substrate level phosphorylation (mSLP) in the glutamine-driven glutaminolysis pathway, substantiated by the succinate-CoA ligase reaction in the TCA cycle, can partially compensate for reduced ATP synthesis through both OxPhos and glycolysis. A protracted insufficiency of OxPhos coupled with elevated glycolysis and an auxiliary, fully operational mSLP, would cause a cell to enter its default state of unbridled proliferation with consequent dedifferentiation and apoptotic resistance, i.e., cancer. The simultaneous restriction of glucose and glutamine offers a therapeutic strategy for managing cancer.

## Graphical Abstract

**Figure:**
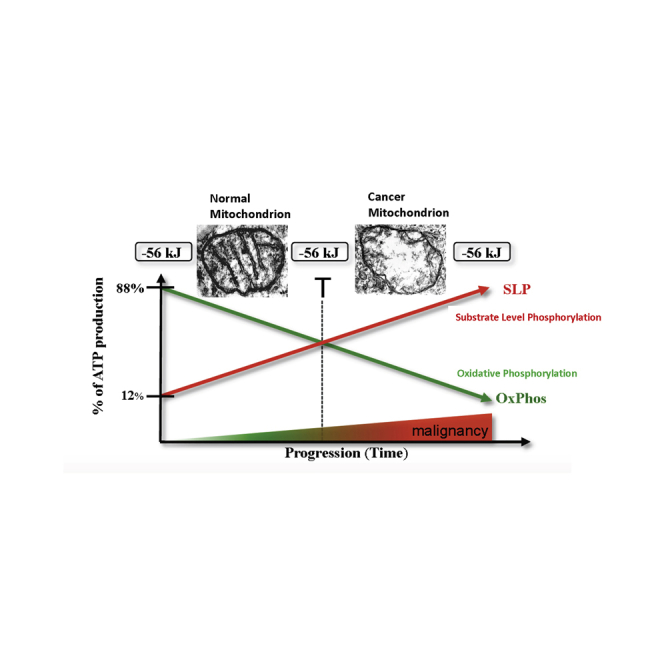

## Introduction

Energy is necessary for cell viability. Most energy is stored in the terminal γ and β phosphates of ATP and is released during the hydrolysis of the phosphoanhydride bonds. This energy is generally referred to as the free energy of activation or ATP hydrolysis (Seyfried, 2012b; Veech et al., 2019b; Harold, 1986; Kocherginsky, 2009). The standard energy of ATP hydrolysis under physiological conditions is known as ΔG′_ATP_ and is tightly regulated in all cells between −53 and −60 kJ/mol (Veech et al., 1979, 2019b). ΔG′_ATP_ differs from the ΔG^0^′_ATP_, which represents the free energy of activation under closed conditions where temperature, gases, and solutes are all standardized. The ΔG′_ATP_ relates more to the situation in open systems, i.e., the situation in cells and tissues (Seyfried, 2012b; Veech et al., 2001, 2019b; Schneider and Sagan, 2005; Banks and Vernon, 1970). A remarkable finding was the similarity of the ΔG′_ATP_ among cells with widely differing resting membrane potentials and mechanisms of energy production. For example, the ΔG′_ATP_ in heart, liver, and erythrocytes was approximately −56 kJ/mol despite having very different electrical potentials of −86, −56, and −6 mV, respectively (Veech et al., 2019a, 2019b). Moreover, energy production in the heart and liver, which contain many mitochondria, is largely through OxPhos, whereas energy production in the erythrocyte, which contains no nucleus or mitochondria, is entirely through glycolysis. Despite the profound differences in resting membrane potentials and in mechanisms of energy production among these disparate cell types, they all express a similar free energy of ATP hydrolysis. These observations suggest that the balance of energy consumption and production is independent of energy source and the amount of total ATP produced.

The constancy of the ΔG′_ATP_ of approximately −56 kJ/mol is fundamental to cellular energy homeostasis, and its relationship to cancer cell energy metabolism is critical. Veech refers to this energy value as the “the still point of the turning world” (Veech et al., 2019b). Why this particular free energy of ATP hydrolysis is so important for cell physiology remains unclear (Hochachka and Somero, 2002; Veech et al., 2019b). Nevertheless, the maintenance of the ΔG′_ATP_ is the endpoint of both genetic and metabolic processes, and any disturbance in this energy balance will compromise cell function and viability (Veech et al., 2001, 2019b). Although the free energy of ATP hydrolysis is used to power nearly all cellular activities, the majority of energy in any given cell is used to power ionic membrane pumps (Veech et al., 2001, 2019a; Seyfried, 2012b; Hochachka and Somero, 2002; Masuda et al., 2005; Harold, 1986; Seyfried and Mukherjee, 2005). If energy to the cellular pumps is interrupted, the cell begins to swell. Swelling results from increased Na^+^ and Ca^2+^ concentration and decreased K^+^ concentration. As the inside of the cell is more negative than the outside, Na^+^ and Ca^2+^ will naturally move down their concentration gradient from outside to inside. On the other hand, K^+^, which is more concentrated inside than outside, will flow down its concentration gradient. Most cell functions are linked either directly or indirectly to the plasma membrane potential and to the Na^+^/K^+^/Ca^2+^ gradients (Seyfried, 2012b; Veech et al., 2019a). Ready availability of ATP to the pumps maintains these ionic gradients and cell viability. Global cellular dysfunction and ultimately organ and systems failure will arise if energy flow to the pumps is disrupted. Hence, chemical energy by itself is the central issue for cell viability.

There are several sources of ATP synthesis that can be used to maintain membrane potentials. The mitochondria produce most energy in normal mammalian cells. The ATP is derived mostly from oxidative phosphorylation (OxPhos) where approximately 89% of total cellular energy is produced (about 32/36 total ATP molecules during the complete oxidation of glucose). This value can differ among different cells depending on which shuttle systems are used in the transport of cytoplasmic reducing equivalents (nicotinamide adenine dinucleotide, NADH) from the cytoplasm to the mitochondria (Seyfried, 2012b). These shuttles primarily include the malate-aspartate shuttle, and the glycerol-phosphate shuttle. Although operational in tumor cells, the activity of these shuttles can differ among the different types of tumor cells (Chiaretti et al., 1979; Grivell et al., 1995; Greenhouse and Lehninger, 1976, 1977; Moreadith and Lehninger, 1984; Mazurek et al., 1997, 2005). Under OxPhos, ATP synthesis in normal cells is coupled to electron flow across the inner mitochondrial membrane through a chemiosmotic molecular mechanism.

The F_o_F_1_-ATPase (Complex V), generates ATP through condensation of ADP and inorganic phosphate Pi. Oxygen is the final acceptor of electrons with water as the end product (Seyfried, 2012b). The efficiency of the process is dependent in large part on the lipid composition of the inner mitochondrial membrane where cardiolipin is a major component, and on structural integrity of the cristae (Kiebish et al., 2008; Cogliati et al., 2016). The proton motive gradient of the inner mitochondrial membrane, symbolized as ΔΨm, is required not only for ATP synthesis but also for transport functions including those for nucleotides, amino acids, Ca^2+^, and other metabolites needed for normal mitochondrial function. The maintenance of this gradient is essential for normal mitochondrial function and ultimately, cell function and life (Brand and Nicholls, 2011; Veech et al., 2019b; Hochachka and Somero, 2002; Schneider and Sagan, 2005). Galluzzi and colleagues provide a more complete coverage of the multiple functions of mitochondria and discuss how these functions can be the gateway to tumorigenesis (Galluzzi et al., 2010). Besides OxPhos, approximately 11% (4/36 total ATP molecules) of total cellular energy is produced through substrate-level phosphorylation (SLP) reactions in the cytoplasm (2 ATP) and mitochondria (2 ATP). SLP involves transfer of a phosphate to ADP from a metabolic substrate to form ATP. Two major metabolic pathways can produce ATP through SLP in mammalian cells and tissues. The first involves the “pay off” part of the Embden-Myerhoff-Parnas glycolytic pathway in the cytosol where phosphate groups are transferred from the organic molecules, 1,3-bisphosphoglycerate and phosphoenolpyruvate, to ADP with formation of ATP. The second pathway involves the succinyl-CoA ligase (SUCL) reaction of the tricarboxylic acid (TCA) cycle. The coordination of ATP synthesis through OxPhos and the SLP reactions underlies metabolic homeostasis that is essential for maintaining cellular differentiation.

## Mitochondrial Network and Mitochondria-Associated Membrane Abnormalities: A General Phenotype of Cancer

**Figure 1:**
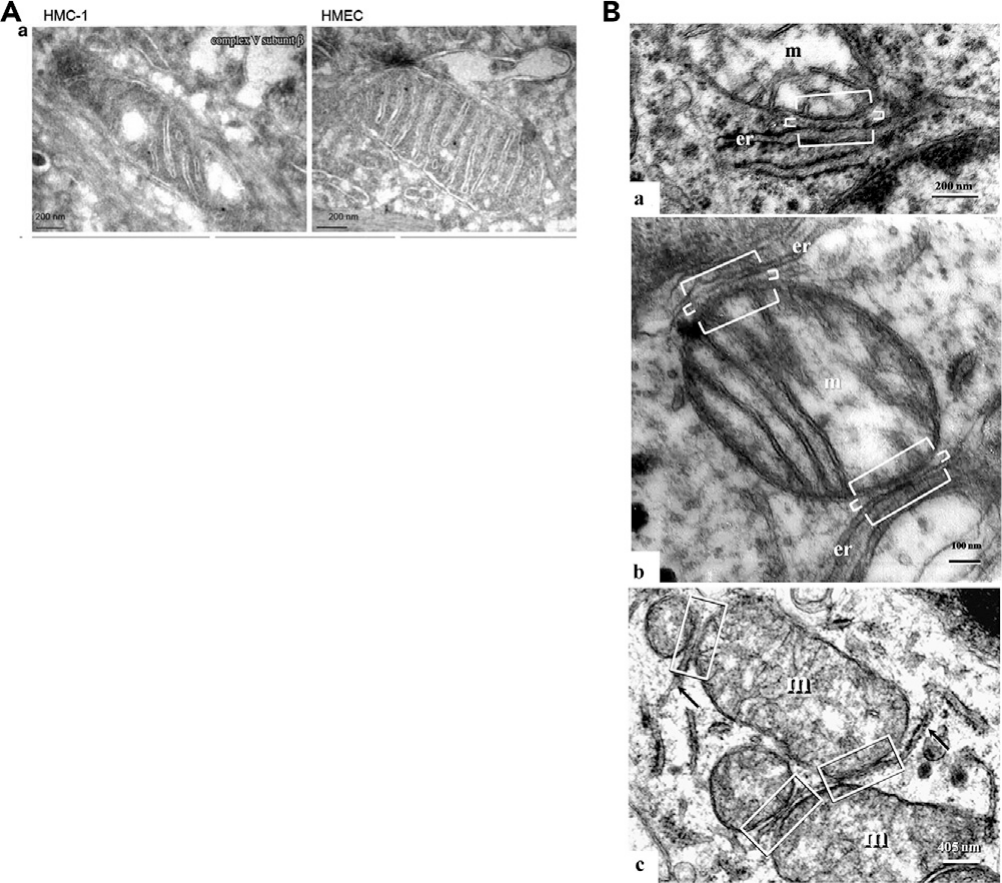
Ultrastructral Abnormalities in Cancer Mitochondria (A) Immunogold electron microscopy on ultrathin cryosections of breast cancer specimen primary cell culture (human mammary carcinoma HMC-1) and human epithelial mammary cell reference line (HEMC). Mitochondrial morphology was clearly deteriorated in the HMC-1 cell with loss of invaginations and vacuolization of the sub-mitochondrial structure. These abnormalities in mitochondria ultrastructure were also associated with abnormalities in the electron transport chain and are in general agreement with those from other studies of breast cancer mitochondria (Roddy and Silverberg, 1980; Elliott et al., 2012; Jogalekar and Serrano, 2018). Reprinted with permission from (Putignani et al., 2008). (B) Ultrastructural abnormalities in mitochondria and mitochondrial-associated membranes (MAM) of human glioblastoma. Micrographs show mitochondria with disarrangement of cristae and partial or total cristolysis, and condensed configuration. These structural abnormalities in the mitochondrial network would reduce ATP synthesis through OxPhos, thus requiring compensatory ATP synthesis through substrate level phosphorylation both in the cytoplasm using glucose as substrate (Warburg effect) and in the mitochondria using glutamine as substrate (Q-Effect) (see text for details). The MAM anomalies found in gliomas have also been observed in other cancers and are linked to abnormalities in calcium homeostasis, proliferation, metastasis, and chemotherapeutic resistance. (a) MAM length 320 nm (long brackets). Mitochondria-ER interface <30 nm (direct association) (short brackets); m denotes electron-lucent mitochondria with partial cristolysis and er denotes non-expanded ER profiles. (b) MAM length 258.3–287.5 nm (long brackets). Mitochondria-ER interface <30 nm (direct association) (short brackets); m denotes electron-lucent mitochondria with partial cristolysis and er denotes non-expanded ER profiles. (c) MAM length 96–652 nm, and mitochondria-ER interface <30 nm (direct association) (rectangle); m denotes electron-lucent mitochondria with total cristolysis and er denotes non-expanded ER profiles. Dense mitochondria (m), dilated ER profiles (er), and MAM displaying direct associations (interface ≤30 nm) (brackets) are observed. Magnifications: ×5.000–35,000. Reprinted with permission from (Arismendi-Morillo et al., 2017).

Mitochondrial function is closely linked to mitochondrial structure and the efficiency of OxPhos (Lehninger, 1964; Stroud and Ryan, 2013; Hackenbrock, 1968; Putignani et al., 2012; Cogliati et al., 2016; Brand and Nicholls, 2011). Abnormalities in mitochondrial number, ultrastructure, and function have been documented in all major cancers (Table 1). The mitochondrial network exhibits heterogeneous ultrastructural pathology in many human tumors (Arismendi-Morillo et al., 2017; Morciano et al., 2018; Pagano et al., 2014; Pedersen, 1978; Arismendi-Morillo, 2009; Simoes et al., 2020). This heterogeneous pathology involves abnormalities in number of mitochondria, structural abnormalities in mitochondrial cristae, alterations in mitochondrial lipids and enzymes of the electron transport chain (ETC), and abnormalities in mitochondrial-associated membranes (MAM). As MAM are intimately associated with mitochondrial function, alterations in MAM structure will alter mitochondrial function and reduce the efficiency of OxPhos (Arismendi-Morillo et al., 2017; Simoes et al., 2020). Examples of mitochondrial and MAM ultrastructural abnormalities in breast cancer and glioblastoma are illustrated in Figures 1A and 1B. A high cristae surface area is predicted to favor ATP synthesis (Quintana-Cabrera et al., 2018). Mitochondria with partial or total cristolysis predominate in malignant tumors making OxPhos inefficient. Perturbations of mitochondrial-shaping proteins disrupt cristae organization making the ETC less efficient thereby decreasing the efficiency of OxPhos (Cogliati et al., 2016). Alterations in the density, length, and width of the MAM and MAM-resident mTORC2 would increase reactive oxygen species (ROS) production, thus causing a metabolic shift from energy production through OxPhos to energy production through SLP in the glycolysis and glutaminolysis pathways. Abnormalities in MAM ultrastructure have been found in cancer tissue that involve the density, the length, and the width of the interfacing membranes of the mitochondria and endoplasmic reticulum (ER) (Arismendi-Morillo, 2009; Arismendi-Morillo et al., 2017; Simoes et al., 2020). The ultrastructural abnormalities in mitochondria and MAM represent the submicroscopic base for abnormal cancer metabolism leading to a greater reliance on SLP than on OxPhos for energy production. The mitochondrial and MAM morphological abnormalities are also dependent on the tumor microenvironment and are not specific for any tumor type.

**Table 1:** Evidence for Abnormalities in Mitochondrial Number, Structure, or Function in Various Cancers

**Figure 2:**
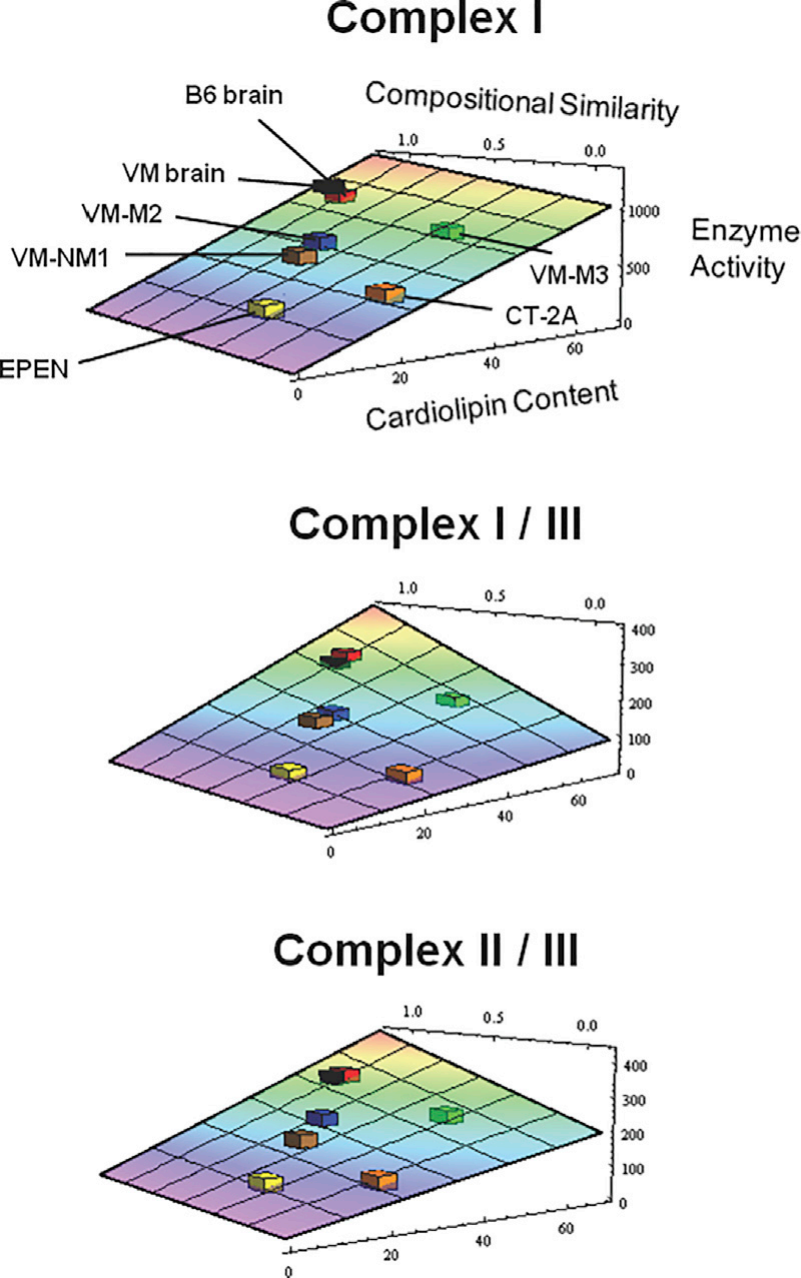
Relationship of Abnormalities in Cardiolipin Content and Composition to Abnormalities in Electron Transport Chain Activities in Mouse Brain Tumors Data were derived from brain tumors grown in the C57BL/6 (B6) and the VM/Dk (VM) inbred mouse strains. The best-fit three-dimensional quadratic surface was used to express data for each electron transport chain (ETC) complex, as described previously (Kiebish et al., 2008). The data for the VM/Dk strain and VM-derived tumors were fit to the C57BL/6-fit quadratic surface to illustrate the position of all tumors on the same graph relative to their host mouse strains. As the ETC complexes I, I/III, and II/III are necessary for maintaining the mitochondrial proton gradient and energy production through OxPhos (Haines and Dencher, 2002; Genova et al., 2003), the CL abnormalities found in these brain tumors will compromise efficiency of respiratory energy metabolism. These data show that abnormalities in the fatty acid composition and content of cardiolipin (CL) are linked to abnormalities in the activities of ETC complexes. Reprinted with permission from (Kiebish et al., 2008).

**Bladder cancer:** (Massari et al., 2016; Moriyama et al., 1984; Papadimitriou and Drachenberg, 1994).
**Breast/mammary cancers**: (Elliott et al., 2012; Gadaleanu and Craciun, 1987; Guha et al., 2018; Guo et al., 2018; Jogalekar and Serrano, 2018; Ma et al., 2010; Morciano et al., 2018; Owens et al., 2011; Pagano et al., 2014; Putignani et al., 2008; Putignani et al., 2012; Roddy and Silverberg, 1980; Roskelley et al., 1943; Rouiller, 1960; Santidrian et al., 2013).
**Colorectal cancers**: (Modica-Napolitano et al., 1989; Piscitelli et al., 2003; Roskelley et al., 1943; Sun et al., 1981),
**Gliomas**: (Arismendi-Morillo and Castellano-Ramirez, 2008; Chinopoulos and Seyfried, 2018; Deighton et al., 2014a; Deighton et al., 2014b; Feichtinger et al., 2014; Katsetos et al., 2013; Oudard et al., 1997; Scheithauer and Bruner, 1987; Seyfried et al., 2019; Sipe et al., 1973).
**Kidney/renal cancer**: (Moreno et al., 2005; Roskelley et al., 1943; Sarto et al., 1997; Simonnet et al., 2003; Yusenko et al., 2010).
**Leukemias/lymphoma including AML and ALL**: (Huhn, 1984; Huhn et al., 1984; Kluza et al., 2011; Morciano et al., 2018; Roskelley et al., 1943; Schumacher et al., 1975; Schumacher et al., 1974).
**Liver/hepatic cancer:** (Capuano et al., 1996; Cheuk and Chan, 2001; Cuezva et al., 2002; Cuezva et al., 2009; Lo et al., 1968; Pedersen, 1978; Rouiller, 1960; Volman, 1978; White et al., 1974).
**Lung cancer**: (Fernandez et al., 1976; Momcilovic et al., 2019; Morciano et al., 2018; Nicolescu and Eskenasy, 1984a, 1984b).
**Melanomas**: (Hall et al., 2013; Taddei et al., 2012; White et al., 1974),
**Neuroblastoma**: (Brawn and Mackay, 1980; Feichtinger et al., 2010; Morscher et al., 2015).
**Osteosarcoma**: (Friedman and Gold, 1968; Ghadially and Mehta, 1970; Hou-Jensen et al., 1972; Van Waveren et al., 2006).
**Ovarian cancer**: (Andrews and Albright, 1992; Dai et al., 2010; Ishioka et al., 2004).
**Pancreatic cancer**: (Huntrakoon, 1983; Legrand and Pariente, 1976; Novotny et al., 2013).
**Prostate cancer**: (Mao et al., 1966; Roskelley et al., 1943; Vayalil and Landar, 2015).
**Rhabdomyosacromas:** (Bundtzen and Norback, 1982; Li et al., 2018).
**Retinoblastoma:** (Singh et al., 2015; Sun, 1976).
**Salivary gland/oral cancers**: (Kataoka et al., 1991; Kummoona et al., 2008),

In addition to the ultrastructural abnormalities in mitochondria and MAM, no cancer cell has been found with a normal content or composition of cardiolipin, the cristae-enriched phospholipid that contributes to OxPhos function (Kiebish et al., 2008; Chicco and Sparagna, 2007; Claypool et al., 2008; Cogliati et al., 2016). Cardiolipin is recognized as essential for the proper function of ETC supercomplex structures, which are linked directly to cristae ultrastructure (Guo et al., 2018). Figure 2 illustrates the linkage of cardiolipin abnormalities to abnormalities in ETC enzyme activities in five syngeneic mouse brain tumors. ETC supercomplex structures were found to be abnormal in these mouse tumors. It is also interesting that the ETC defects found in these mouse tumors did not arise from mutations within the mitochondrial genome, as no pathogenic mutations were found in the sequenced mitochondrial genome of each tumor (Kiebish and Seyfried, 2005). However, pathogenic mutations that could disrupt mitochondrial function have been found in other tumors (Gammage and Frezza, 2019; Yuan et al., 2020; Cruz-Bermudez et al., 2015). Although some gene mutations can cause abnormalities in mitochondrial structure and function, mitochondrial abnormalities can also arise independently of gene mutations (Seyfried et al., 2020; Seyfried, 2012a). Proton leak and uncoupling, which diminish respiratory efficiency (Brand and Nicholls, 2011), are also greater in tumor cells than in normal cells (Seyfried, 2012e, Villalobo and Lehninger, 1979; Lemarie and Grimm, 2011). Based on the foundational biological principle that *structure determines function* (Lehninger, 1964; Chinopoulos and Seyfried, 2018; Mayer, 1982; Putignani et al., 2012; Cogliati et al., 2016), abnormalities in mitochondria structure would alter mitochondria function and effective ATP synthesis through OxPhos. How could ATP synthesis through OxPhos be considered normal in any tumor cell where the very structures needed for normal OxPhos function are abnormal? Energy through OxPhos is dependent on the integrity of mitochondrial structure and function.

Besides these documented abnormalities in mitochondria structure and function, genetic abnormalities that alter mitochondrial function have also been recognized in many cancers. The *p53* mutation, which is found in many cancers, can disrupt mitochondrial OxPhos (Zhou et al., 2003; Bartesaghi et al., 2015; Matoba et al., 2006). The retinoblastoma tumor suppressor protein, Rb, has been linked to abnormalities in mitochondrial mass and OxPhos function (Nicolay et al., 2015). Abnormalities in mitochondrial structure or function have also been associated with other cancer-related genes including *BCR-ABL* (Capala et al., 2016), the *V600E-BRAF* oncogene (Hall et al., 2013), and *BRCA* mutations (Chung et al., 2017; Henderson, 2012; Maniccia et al., 2009; Privat et al., 2014). Huang and colleagues showed that *K-rasG12v* transformation caused mitochondrial dysfunction and a metabolic switch from OxPhos to aerobic fermentation (Hu et al., 2012). None of the mentioned genetic abnormalities are 100% penetrant and are therefore considered secondary causes (risk factors) rather than primary causes of cancer. It appears that few if any cancer types are free of mitochondrial abnormalities, whether structural or functional, making OxPhos inefficiency the signature metabolic hallmark of cancer. As tumor cells require a significant ATP/ADP ratio for invasion (Zhang et al., 2019), an alternative system for ATP synthesis must be in place to compensate for OxPhos inefficiency. A reliance on cytoplasmic and mitochondrial SLP can provide both the necessary ATP and the metabolic building blocks needed for tumor cell proliferation and invasion in either aerobic or anaerobic growth environments.

## Is ATP Synthesis from Both Fermentation and OxPhos Necessary to Meet Tumor Cell Energy Demands?

Despite the extensive evidence reviewed above describing mitochondrial anomalies in various cancers, many investigators have claimed that mitochondria and OxPhos are intact or not seriously compromised in some cancer cells. Warburg also linked oxygen consumption to ATP synthesis through OxPhos in tumor cells despite his view that mitochondrial respiration was abnormal in cancer (Warburg et al., 1927; Warburg, 1956b). Consideration of normal mitochondrial function in cancer originated with Sidney Weinhouse who indicated that oxygen consumption and P/O ratios can be similar in normal cells and tumor cells, suggesting that tumor cells require both fermentation and OxPhos energy to meet energy demands (Weinhouse, 1956, 1976). The Weinhouse opinion has been cited by some investigators as evidence against Warburg's central theory that OxPhos dysfunction is the origin of cancer (Koppenol et al., 2011; Vander Heiden et al., 2009; Vaupel, 2008; Weinberg et al., 2010; Sun et al., 2019). The P/O ratio (mol of ATP synthesized per mol of oxygen used); however, is not a good indicator of mitochondrial function especially in cancer cells (Brand and Nicholls, 2011; Warburg, 1956a, 1956b; Burk and Schade, 1956). The Weinhouse group also published data documenting mitochondrial abnormalities in hepatomas (Lo et al., 1968). Specifically, they found that the content of the mitochondrial-enriched beta-hydroxybutyrate dehydrogenase was significantly lower in poorly differentiated tumors (9–14 mg/g tissue) than in normal liver mitochondria (50 mg/g) or in well differentiated tumors (18–33 mg/g). They concluded that high respiration is characteristic of normal liver cells and of well-differentiated hepatomas, and that low respiration, coupled with loss of mitochondria, accompanied loss of differentiation (Lo et al., 1968; Morris et al., 1960). Although Weinhouse first mentioned that fatty acid metabolism to CO_2_ was similar in tumors and in normal tissues, his group later showed that fatty acids could not be used for ATP synthesis in highly glycolytic hepatomas despite producing normal CO_2_ levels (Bloch-Frankenthal et al., 1965; Weinhouse, 1956). More recent studies have confirmed observations that tumor cells produce little ATP from fatty acids (Ta and Seyfried, 2015; Kuok et al., 2019; Lin et al., 2017). It is important to mention that much of the evidence supporting the view that OxPhos is intact or is not seriously compromised in cancer cells comes from *in vitro* studies. For example, OxPhos function obtained from cultured MCF7 and MDA-MB-231 breast cancer cells, which appeared mostly normal (LeBleu et al., 2014), was inconsistent with the abnormal OxPhos function seen in clinical breast cancer tissue (Koit et al., 2017). Data obtained from *in vitro* studies must therefore be viewed cautiously in light of the documented mitochondrial abnormalities seen in the intact tumor tissues listed in Table 1 and discussed by others (Momcilovic et al., 2019; Burk and Schade, 1956; Hall et al., 2013). Additionally, there can be a potential “hijacking” of the ETC, manifesting as high rates of oxygen consumption, without a corresponding synthesis of ATP through OxPhos (reviewed below). Hence, information on OxPhos function in cancer cells is likely to be more accurate when *in vitro* data are consistent with *in vivo* observations.

**Figure 3:**
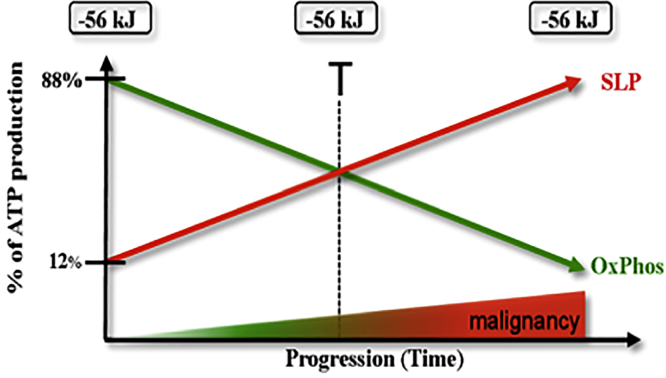
Transition of ATP Synthesis from OxPhos to SLP in the Origin and Progression of Cancer Cancer can arise from any number of non-specific events that damage the respiratory capacity of cells over time, as described previously (Seyfried and Shelton, 2010; Seyfried et al., 2014). The path to carcinogenesis will occur only in those cells capable of enhancing energy production through SLP. Despite the gradual shift from OxPhos to SLP, the ΔG′ of ATP hydrolysis will remain fairly constant at approximately −56 kJ, indicating that ATP synthesis through SLP compensates for the reduced energy from OxPhos. The mitochondrial stress response will initiate oncogene up-regulation that becomes necessary for importing increased amounts of the glucose and the glutamine needed for the synthesis of growth metabolites and ATP through SLP (Seyfried, 2012f; Seyfried et al., 2014). Genomic instability and somatic mutations would arise as a consequence of protracted mitochondrial ROS production together with acidification of extracellular microenvironment (Seyfried et al., 2014). Metastasis arises from respiratory damage in cells of myeloid/macrophage origin that would use glutamine as a major fuel for ATP synthesis (Seyfried and Huysentruyt, 2013; Seyfried et al., 2020). The degree of tumor progression and malignancy can be linked directly to the energy transition from OxPhos to SLP. This scenario was shown to connect all major cancer hallmarks to an extrachromosomal respiratory dysfunction (Seyfried et al., 2014; Seyfried, 2012d). The T signifies an arbitrary threshold when the transition from OxPhos to SLP becomes irreversible. Although collections of ROS-induced somatic mutations would contribute to disease irreversibility, the mutations can also prevent metabolic adaption to glucose and glutamine targeting (see Figure 5) (Seyfried and Mukherjee, 2005). Reprinted with modifications from (Seyfried and Shelton, 2010; Seyfried et al., 2014).

Diminished OxPhos, coupled with increased SLP activities, can maintain an adequate ΔG′_ATP_ hydrolysis for cell viability during the gradual transition from the differentiated OxPhos-dependent state to the fully malignant SLP-dependent state (Chinopoulos and Seyfried, 2018). Figure 3 shows the constancy of the ΔG′_ATP_ hydrolysis of −56kJ/mole during the transition in ATP synthesis from OxPhos to SLP. Hence, the ΔG′_ATP_ hydrolysis of −56kJ/mole is linked more to cell viability than to cell differentiation. Further consideration of these concepts related to cancer cell energy metabolism is warranted.

## Is Oxygen Consumption Linked to ATP Synthesis through OxPhos in Cancer Cells?

The linkage of oxygen consumption to ATP synthesis through OxPhos has been well established for cells in normal tissues, whereas the linkage of oxygen consumption to OxPhos function has been ambiguous at best in cancer cells (Pacini and Borziani, 2016; Leznev et al., 2013; Hall et al., 2013; Ramanathan et al., 2005; Arcos et al., 1969; Velez et al., 2013). Cells using oxygen consumption for ATP synthesis will die quickly under hypoxia or when treated with cyanide. As many cancer cells can survive when treated with cyanide or in hypoxia, ATP synthesis in these cells must come from sources other than OxPhos (Warburg, 1931; Ta and Seyfried, 2015; Warburg et al., 1927; Barron, 1930; Renner et al., 2010; Ceruti et al., 2005; Seyfried, 2012c). Although Warburg and colleagues found that oxygen consumption could be similar in normal cells and some tumor cells, it was recognized that oxygen consumption was largely disconnected from ATP synthesis in tumor cells (Warburg, 1956a, 1956b; Burk and Schade, 1956). How could OxPhos be impaired in tumor cells if oxygen consumption rates were similar in normal cells and tumor cells? Ramanathan et al. stated it was “*intriguing that cells with the highest tumorigenic potential consumed more oxygen and yet exhibit diminished oxygen dependent (aerobic) ATP synthesis*” (Ramanathan et al., 2005). The authors considered that such tumor cells used the mitochondrial ETC for reasons other than ATP synthesis by allowing leakage of the membrane potential thus producing heat and ROS. Indeed, heat production is greater in cancer cells than in non-cancerous cells, consistent with mitochondrial uncoupling (van Wijk et al., 1984; Gautherie, 1980). ROS production is also greater in cancer cells than in normal cells (Chen et al., 2016; Szatrowski and Nathan, 1991; Aykin-Burns et al., 2009; Zhou et al., 2014; Ayyasamy et al., 2011; Bartesaghi et al., 2015). The genomic instability and random somatic mutations seen in most cancers arise largely as downstream epiphenomenon of ROS production and OxPhos dysfunction (Fosslien, 2008; Bartesaghi et al., 2015; Galadari et al., 2017; Desler et al., 2010; Degtyareva et al., 2013; Seyfried et al., 2020). Furthermore, mitochondrial oxygen consumption increases, rather than decreases, during the S phase of the cell cycle in some cancer cells without generation of ATP further indicating that oxygen consumption was not connected to ATP synthesis through OxPhos (Olivotto et al., 1984). de Groof et al. also showed that increased oxygen consumption was linked directly to mitochondrial abnormalities and increased ROS production following *H-RasV12/E1A*-induced transformation (de Groof et al., 2009). Pacini and Borziani cited numerous studies showing that the dry measure of oxygen consumption is a poor method for determining ATP production through the mitochondrial pathway in cancer cells (Pacini and Borziani, 2016). Methylene blue increases oxygen consumption in a broad range of cancer cells and tissues, but does not increase oxygen consumption in normal cells or tissues (Barron, 1930). These findings were considered evidence that OxPhos was deficient in tumor cells despite evidence of robust oxygen consumption. These observations would also question the validity of the Meyerhof quotient as an accurate measure of OxPhos function in cancer cells (Koppenol et al., 2011; Weinhouse, 1956; Aisenberg, 1961). Although Warburg's calculations of diminished O_2_ consumption in tumor tissue slices were questioned based on tissue slice thickness (Koppenol et al., 2011), his findings were validated in tissue slice homogenates thus linking diminished O_2_ consumption to diminished respiration (Mayer, 1944).

Additional confusion regarding OxPhos function in cultured cancer cells can come from findings that oxygen consumption rate (OCR), using the *Seahorse* instrument, is linked to ATP synthesis through OxPhos. This instrument, however, can only infer that ATP flux is linked to OCR. As the *Seahorse* instrument is not yet capable of distinguishing ATP synthesis from mSLP or OxPhos, caution is necessary in attempting to link OCR to ATP synthesis through OxPhos in cultured cancer cells using this instrument.

Ramanathan and co-workers suggested that cancer cells exhibit a “hijacked” ETC for the purpose of pyrimidine provision that is required for DNA synthesis (Ramanathan et al., 2005). Orotate is needed for synthesis of pyrimidines, which is produced from dihydroorotate by dihydroorotate dehydrogenase (DOHDH) (Desler et al., 2010). DOHDH is an ETC mitochondrial enzyme that reduces quinones to quinols. This reduction promotes electron flux to complex III and subsequently complex IV, provided that there is oxygen available as a final electron acceptor. The implication of these considerations is that the high OCRs seen in some cancer are linked more to orotate production and DNA synthesis than for ATP synthesis through OxPhos. Indeed, DOHDH inhibitors hold promise as chemotherapeutic agents (He et al., 2014; Koundinya et al., 2018; Li et al., 2019). A possible limitation, however, would be under hypoxia conditions (Desler et al., 2010). The high OCRs observed in many cancers could explain in part why uncoupling is so prevalent in tumors (higher electron flow from DOHDH to complex III and complex IV). These considerations could account for *in vitro* and *in vivo* observations that complexes I and II and the ATP synthase are downregulated in many cancers, whereas complexes III and IV are upregulated (Kiebish et al., 2009; Santidrian et al., 2013; Solaini et al., 2011; Simonnet et al., 2002). Finally, the concept of ETC “hijacking” is compatible with the ultrastructural abnormalities mitochondrial cristae seen frequently in tumors (Table 1). Evidence for impaired ATP synthesis through OxPhos is stronger when linked to measurements of mitochondrial number, structure, and function than when linked to measurements of OCRs, which can provide misinformation on OxPhos function. Viewed collectively, OCR should not be used alone as a biomarker for ATP synthesis through OxPhos in cultured cancer cells.

## Glutamine-Driven mSLP: An Unrecognized Mechanism for ATP Synthesis in Cancer Cells

As reviewed above, abnormalities in the cancer cell mitochondrial network would reduce OxPhos efficiency, thus forcing the cell to rely more heavily on SLP for ATP synthesis. The succinate-CoA ligase (SUCL) is a mitochondrial matrix enzyme that catalyzes the conversion of succinyl-CoA and ADP (or GDP) to CoA-SH, succinate, and ATP (or GTP) (Johnson et al., 1998). SUCL is the major phosphorylation reaction occurring at the substrate level in the TCA cycle (Kaufman et al., 1953; Hunter and Hixon, 1949). Notably, when the SUCL proceeds toward ATP formation it is termed “**mitochondrial substrate-level phosphorylation**” (mSLP), a process that can yield high-energy phosphates in the absence of oxygen. SUCL is a heterodimer composed of an invariant α-subunit encoded by the *SUCLG1* gene and a substrate-specific β-subunit encoded by either the *SUCLA2* or *SUCLG2* genes. This dimer combination results in either of two reversible enzyme reactions, i.e., a GTP-forming SUCL (EC 6.2.1.4) or an ATP-forming SUCL (EC 6.2.1.5) (Li et al., 2013). Chen recently described how mSLP could compensate in part for lost ATP synthesis through either glycolysis or OxPhos (Chen et al., 2018). Energy generation through mSLP is critically important in several metabolic pathways and could compensate for inefficient energy production through OxPhos in cancer cells (Tretter et al., 2016; Flores et al., 2018).

**Figure 4:**
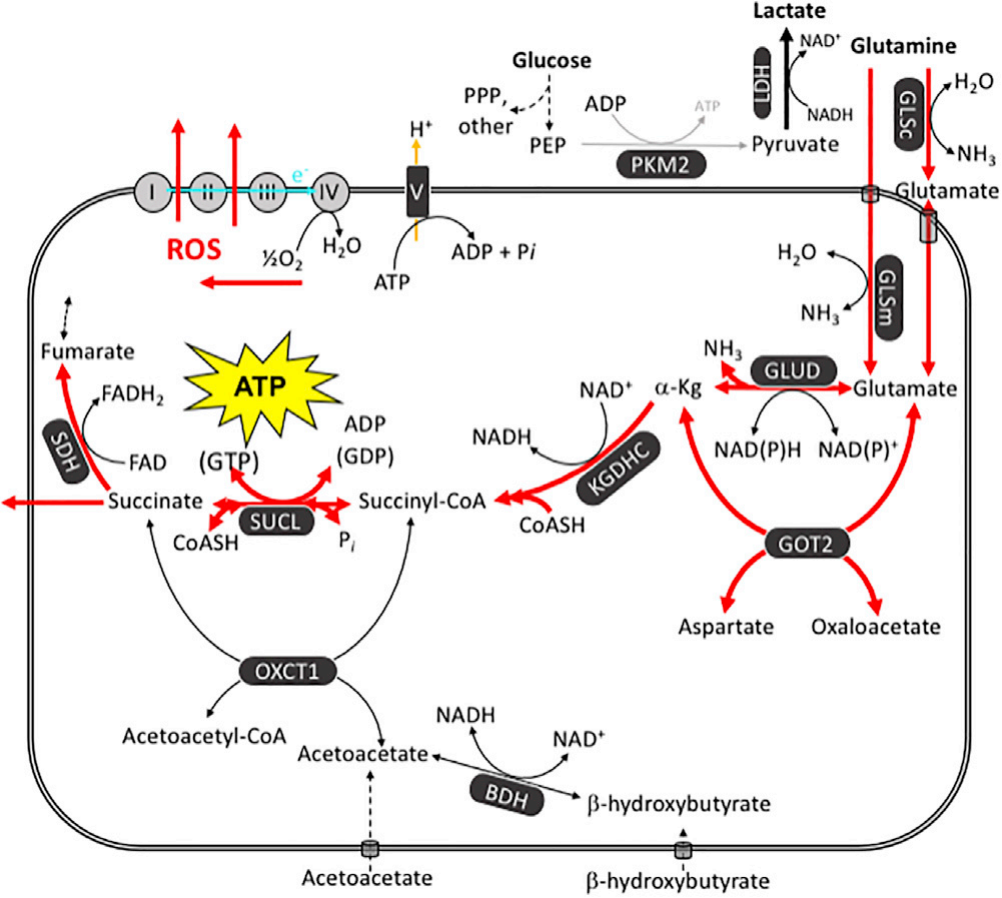
Glutamine-Driven mSLP as a Major Source of ATP Synthesis in Cancer Cells ATP synthesis through mSLP at the succinate-CoA ligase reaction (SUCL) in the glutaminolysis pathway (red) can compensate for inefficient ATP synthesis through OxPhos in cancer cells with mitochondrial abnormalities. mSLP can also compensate for inefficient ATP synthesis through glycolysis in cancer cells that express the cytoplasmic PKM2 isoform, which produces less ATP than the PKM1 isoform. These bioenergetic compensations will hold the ΔG′_ATP_ hydrolysis at minus 56 kJ/mole, thus maintaining cancer cell viability in either the presence or absence of oxygen. Oxygen consumption in cancer cells is used more for production of ROS, which are carcinogenic and mutagenic, than for ATP synthesis. Moreover, mSLP will maintain the forward operation of the adenine nucleotide translocase thus preventing depletion of cytosolic ATP reserves from the reverse operation of the F0-F1 ATP synthase (Chinopoulos and Seyfried, 2018). Release of succinate to the cytoplasm can help stabilize Hif-1a, thus linking lactic acid fermentation through glycolysis to glutamine fermentation through glutaminolysis. The diversion of CoA from succinate to acetoacetate under the metabolism of ketone bodies (β-hydroxybutyrate and acetoacetate) could indirectly reduce ATP synthesis through the SUCL reaction. The simultaneous restriction of glucose and glutamine, while elevating circulating ketone bodies, will stress the majority of signaling pathways necessary for maintaining tumor cell viability (Mukherjee et al., 2019; Chinopoulos and Seyfried, 2018). See text for additional details. BDH, β-hydroxybutyrate dehydrogenase; FAD, flavin adenine dinucleotide; GLSc, glutaminase, cytosolic; GLSm, glutaminase, mitochondrial; GLUD, glutamate dehydrogenase; GOT2, aspartate aminotransferase; KGDHC, α-ketoglutarate dehydrogenase complex; LDH, lactate dehydrogenase; NME, nucleoside diphosphate kinase; OXCT1, succinyl-CoA:3-ketoacid coenzyme A transferase 1; PC, pyruvate carboxylase; PDH, pyruvate dehydrogenase; PEP, phosphoenolpyruvate; PKM2, pyruvate kinase M2; SDH, succinate dehydrogenase; SUCL, succinate-CoA ligase.

Much of the confusion surrounding the issue of OxPhos impairment in cancer arises from a failure to recognize mSLP as another mechanism for ATP synthesis. ATP synthesis through mSLP can be misinterpreted as energy through OxPhos unless experiments are designed to distinguish the two energy sources (Chinopoulos et al., 2019). We recently proposed how the SUCL reaction in the TCA cycle could synthesize ATP (and/or GTP) thus “bailing-in” cancer mitochondria from a reverse-operating F_0_-F_1_ ATP synthase when OxPhos function(s) are impaired (Chinopoulos and Seyfried, 2018). The glutaminolysis pathway would support production of high-energy phosphates through the sequential metabolism of glutamine → glutamate → α-ketoglutarate → succinyl CoA → succinate (Figure 4).

Glutamine has long been considered an essential metabolite for tumor cell growth (Oizel et al., 2017; Nilsson et al., 2020; Still and Yuneva, 2017; Mckeehan, 1982; Dang, 2010). We described how glutamine-derived α-ketoglutarate could branch out to become a substrate for both the reductive carboxylation and the oxidative decarboxylation pathways in the TCA cycle (Chinopoulos and Seyfried, 2018). ATP synthesis through mSLP has been recognized in cardiac and kidney tissues under hypoxia (Weinberg et al., 2000; Gronow and Cohen, 1984; Pisarenko et al., 1985), and in cells with mitochondrial DNA (mtDNA) mutations (Chen et al., 2018). mSLP could also rescue respiratory growth in yeast with deficiency in the ATP synthase (Schwimmer et al., 2005). Moreover, OxPhos cannot be normal in cells containing numerous mtDNA mutations, as was recently described for many cancers (Yuan et al., 2020). Compensatory glutamine-dependent mSLP would be an expected consequence of any mtDNA mutation that would compromise OxPhos efficiency. Recent studies also show that mitochondrial import of the ARHGAP11B protein facilitates proliferation of basal progenitor cells through mSLP and glutaminolysis during early brain development (Namba et al., 2019). This is interesting, as emerging evidence shows that glutaminolysis and, as an extension, mSLP, can also serve as a source of ATP synthesis in proliferating cancer cells (Flores et al., 2018; Gao et al., 2016). Chen et al. showed that glutamine utilization is a common feature of cells with partial defects in OxPhos, irrespective of the specific OxPhos complex affected (Chen et al., 2018). OxPhos inefficiency could account in large part for the glutamine addiction of cancers (Chinopoulos and Seyfried, 2018; Still and Yuneva, 2017). Glutamine-supported mSLP can compensate for OxPhos deficiency in either hypoxic or normoxic growth environments. The notion that NAD+ levels are insufficient under hypoxia for supporting KGDHC activity through glutaminolysis is not true, as there are several mechanisms by which NADH can be oxidized to NAD^+^ in the absence of oxygen (Chinopoulos, 2020). Support for this fact comes from findings that robust glutaminolysis occurs in OxPhos-deficient normal cells and in many cancer cells.

Wallace Mckeehan first described glutaminolysis as the second major energy pathway in cancer cells (Mckeehan, 1982). Although Mckeehan recognized the SUCL reaction in the TCA cycle, he did not consider this reaction as a major source of ATP synthesis, but rather considered glutamine as a respiratory fuel for OxPhos, as have other investigators (Mckeehan, 1982; Dang, 2010). However, the uncoupling action of glutamine in metastatic tumor cells could reduce ATP synthesis through OxPhos despite increased oxygen consumption (Hurtaud et al., 2007, and see below). Circumstantial evidence exists supporting the notion that succinyl-CoA (the substrate for mSLP) is formed and metabolized in tumor cells, at least in gliomas (Hadjipanayis et al., 2015). This evidence comes from contrasting neoplastic glioma tissue from normal brain tissue using 5-aminolevulinic acid (5-ALA), which is catabolized to fluorescent porphyrins. Gliomas exhibit an unusual avidity for 5-ALA and express the enzymes for its further metabolism. As 5-ALA originates from the condensation of succinyl-CoA and glycine, it is reasonable to assume that enzymes (such as SUCL) catabolizing succinyl-CoA must be present in tumor tissues. On the other hand, there are tumors devoid of a SUCLA2 due to its proximity to the *RB1* gene, which is frequently deleted in some tumors of the prostate (Kohno et al., 2020). However, such tumors exhibit a pharmacologically targetable vulnerability, which is not present in those cells that do not harbor the SUCLA2 deletion. It should be recognized that abolition of mSLP alone does not hold the key for combating neoplasia. Indeed, a cancer cell dependency score of 5%–11% was obtained for SUCL subunits from cancer dependency analysis (DepMap) (Tsherniak et al., 2017), suggesting that this enzyme might not be critically important for cancer cell survival. As tumor cells rely on both glutaminolysis and glycolysis for growth and survival, it is the simultaneous blocking of both pathways that will confer the greatest effect on tumor viability.

McKeehan considered malate-derived pyruvate as the end product of the glutaminolysis pathway, whereas Moreadith and Lehninger showed that pyruvate was not the end product, as malate did not leave the mitochondria in five different tumor types (Moreadith and Lehninger, 1984). The McKeehan view that pyruvate was the end product of glutaminolysis led to suggestions that significant lactic acid could also be produced from glutamine in cancer cells (DeBerardinis et al., 2007). Most other studies, however, show that little lactate is produced from glutamine in cancer cells (Portais et al., 1996; Ta and Seyfried, 2015; Reitzer et al., 1979; Scott et al., 2011). If lactate is not the end product, what would be the end product of glutaminolysis?

We consider that succinate, rather than pyruvate/lactate, is the end product of the glutaminolysis pathway (Tretter et al., 2016; Chinopoulos, 2019; Chinopoulos and Seyfried, 2018; Figure 4). This is important as succinate is known to stabilize HIF-1α, a transcription factor that together with c-Myc upregulates pathways necessary for the anaerobic metabolism of glucose and glutamine (Tannahill et al., 2013; Tardito et al., 2015; Tennant et al., 2009; Dang, 2011; Semenza, 2010, 2017). In addition to increasing glutaminolysis, c-Myc also enhances expression of the pyruvate kinase M2 isoform, which produces less ATP than the M1 isoform (see below). This observation indicates that succinic acid fermentation through glutaminolysis would enhance lactic acid fermentation through glycolysis (Warburg effect) (David et al., 2010; Dong et al., 2016; Portais et al., 1996). The *HIF-1*α and *c-Myc* oncogenes enable tumor growth by upregulating glucose and glutamine fermentation pathways. Cells that cannot transition from OxPhos to SLP will die and thus cannot become tumorigenic (Warburg, 1956a). Although the nuclear transfer experiments show that oncogene activation cannot be a cause of cancer (Seyfried, 2015), oncogene activation becomes necessary in enabling the transition of ATP synthesis from OxPhos to SLP during tumorigenesis.

We recently proposed that mSLP was the “missing link” in Warburg's central theory that insufficient OxPhos coupled with compensatory fermentation is the origin of cancer (Chinopoulos and Seyfried, 2018). In addition to cytoplasmic SLP (Warburg effect), mSLP could also compensate for OxPhos deficiency. Direct evidence for this possibility comes from the data of Chen et al., showing that human cells with mtDNA mutations and OxPhos deficiency can rewire glutamine metabolism to obtain energy through mSLP (Chen et al., 2018). As *Q* is the letter designation for glutamine, we have described this phenomenon as the *Q-Effect* to distinguish it from that involving the aerobic fermentation of glucose, i.e., the *Warburg effect* (Chinopoulos and Seyfried, 2018). Both the Warburg effect and the Q-effect arise from compromised OxPhos. The role of glutaminolysis and mSLP in cellular energy metabolism was unknown to Warburg, as this information was discovered only after, or toward the end of his career (Sanadi et al., 1956; Kaufman et al., 1953; Ottaway et al., 1981; Hunter and Hixon, 1949). We originally described this phenomenon as the Warburg Q-Effect, but removed the term *Warburg* from the effect, as Warburg neither described nor envisioned amino acid fermentation as a second major compensatory energy source to OxPhos in his theory on the origin of cancer (Warburg, 1931, 1956a, 1956b, 1969; Warburg et al., 1927). Further studies will be needed to determine the extent of mSLP as an alternative ATP synthesis mechanism in cancer cell growth and metastasis.

Although most investigators have focused on aerobic fermentation (Warburg effect), none of the major review articles or previous studies on cancer energy metabolism have discussed or even recognized the role of SUCL activity and mSLP as an energy mechanism that could compensate for deficient OxPhos in tumor cells (Chinopoulos and Seyfried, 2018; Seyfried, 2012c). This would be especially the case for those tumors expressing the glycolytic pyruvate kinase M2 (PKM2) isoform, which predominates in many aggressive cancers and produces less ATP than the PKM1 isoform (David et al., 2010; Dong et al., 2016; Vander Heiden, 2010; Israelsen et al., 2013; Yu et al., 2019; Mazurek et al., 2005). It was shown that ROS inhibition of PKM2 diverts glycolytic intermediates to the pentose phosphate pathway, thus boosting cellular antioxidant responses (Anastasiou et al., 2011), and, by the same token, meaning that ATP synthesis through glycolysis is also diminished. How could any cancer cell survive and grow if its ATP synthesis was diminished through both OxPhos and glycolysis?

We consider mSLP as the dominant mechanism for ATP synthesis in tumor cells with ultrastructural abnormalities in mitochondrial cristae, that overexpress PKM2, and that grow in hypoxic environments. Chen et al. showed that ATP synthesis through mSLP could compensate for ATP syntheses deficiencies in either glycolysis or OxPhos (Chen et al., 2018). We propose that mSLP is the metabolic hallmark of tumor cell proliferation whether growth is *in vivo* or *in vitro*. The Crabtree effect might induce a similar process in non-tumorigenic cells that proliferate *in vitro*, but would not occur in normal cells that proliferate *in vivo* (Kiebish et al., 2009). Aerobic fermentation does not occur in proliferating non-transformed cells grown *in vivo*, for example, in regenerating liver cells and normal colon cells that use fatty acids and butyrate as respiratory fuels, respectively (Chinopoulos and Seyfried, 2018; Hague et al., 1997; Simek and Sedlacek, 1965; Thevananther, 2010). Warburg also described how aerobic fermentation would confuse the issue of cancer cell metabolism and should not be used as a test for cancer cells (Warburg, 1956b). Support for his position that aerobic fermentation (glycolysis) confuses the issue of cancer metabolism came from the studies of R. J. O'Connor who misinterpreted the linkage of oxygen consumption to cell division in the early chick embryo (O'Connor, 1950; Warburg, 1956a; Warburg, 1956b). It is known that anaerobic fermentation, not aerobic fermentation, is largely responsible for cell division in the early embryo (Warburg, 1956a, 1956b). Confusion over the association of OxPhos to oxygen consumption and a failure to recognize the role of mSLP as a compensatory energy mechanism could explain in large part how some investigators might consider that OxPhos is functional and responsible for ATP synthesis in tumor cells.

## Fatty Acid and Glutamine Stimulation of SLP in Cancer Cells

Previous studies have shown that fatty acids are potent swelling and uncoupling agents that can stimulate insulin secretion and glucose/glutamine consumption, thus making it appear as if tumor cells can metabolize fatty acids for energy (Lehninger, 1964; Vozza et al., 2014; Samudio et al., 2009; Giudetti et al., 2019). The palmitate-induced increase in oxygen consumption, glycolysis, and neutral lipid storage was greater in mitochondria from MDA-231 triple-negative breast cancer cells than from normal cells (Park et al., 2016). Andersen et al. also showed that palmitic acid could enhance glucose uptake in several prostate cancer cell lines suggesting that fatty acids enhance glycolysis through the PI3K/Akt pathway (Andersen et al., 2014). ATP synthesis from fatty acid oxidation occurs only if OxPhos is intact, which is not the case for the majority of cancers. The simultaneous upregulation of glycolysis, glutaminolysis, and oxygen consumption would occur following fatty acid-induced uncoupling of the ETC (Samudio et al., 2009; Valle et al., 2010; Vozza et al., 2014; Chinopoulos and Seyfried, 2018; Rupprecht et al., 2019; Ayyasamy et al., 2011). Such findings might give the impression that tumor cells use fatty acids for energy, as some have suggested. As neither fatty acids nor ketone bodies are fermentable fuels, they cannot replace either glucose or glutamine as the main drivers of cancer growth. Indeed, many tumor cells accumulate fatty acids in the form of cytoplasmic triglyceride lipid drops (Zhang et al., 2016; Giampietri et al., 2017; Hardy et al., 2003; Guo et al., 2013; Bozza and Viola, 2010; Ta and Seyfried, 2015). Triglyceride storage is a means to protect tumor cells from the toxic effects of free fatty acids (Hardy et al., 2003; Listenberger et al., 2003; Ta and Seyfried, 2015). Moreover, triglyceride storage has been linked directly to mitochondrial damage or OxPhos dysfunction (Johnson et al., 2005). As mitochondria structural and functional abnormalities are seen in most major cancers (Table 1), the accumulation of lipid drops in cancer cells can be due in large part to OxPhos insufficiency. These findings are consistent with information from Weinhouse indicating that fatty acids are not used directly for ATP synthesis in malignant hepatomas (Bloch-Frankenthal et al., 1965). Rather, fatty acids can indirectly stimulate tumor cell growth through SLP-linked fermentation mechanisms. Palmitate is also known to increase OCR through its effects on ATP hydrolysis, rather than through ATP synthesis (Kuok et al., 2019). It is also interesting that glutamine alone can uncouple oxygen consumption from ATP synthesis through its effects on uncoupling protein 2 (UCP2) (Hurtaud et al., 2007). This uncoupling mechanism would allow glutamine to generate energy primarily through mSLP rather than through OxPhos. Evidence supporting this mechanism was recently discussed for glioblastoma and for the highly invasive and metastatic VM-M3/Dk murine glioblastoma cells, which share multiple properties with the metastatic RAW264-7 cells (Huysentruyt et al., 2008; Flores et al., 2018; Chinopoulos and Seyfried, 2018; Seyfried and Huysentruyt, 2013). Further studies will be needed to determine how fatty acids and glutamine increase tumor growth through effects on SLP in the glycolysis and the glutaminolysis pathways.

## Targeting Glucose and Glutamine for the Metabolic Management of Cancer

The linkage of fermentation to malignancy is as solid as that of gravity to the redshift (Seyfried et al., 2019; Warburg, 1956b). It is well recognized that most, if not all, tumor cells are dependent on glucose and glutamine for growth (Tardito et al., 2015; Still and Yuneva, 2017; Zielinski et al., 2017; Choi and Park, 2018). Although amino acids other than glutamine can also provide energy through mSLP, glutamine is the only amino acid not requiring expenditure of energy for the metabolic interconversions necessary to produce succinyl-CoA (Chinopoulos and Seyfried, 2018). As the default state of metazoan cells is proliferation, not quiescence (Sonnenschein and Soto, 2000; Soto and Sonnenschein, 2004), unbridled proliferation becomes a consequence when SLP replaces OxPhos for ATP synthesis in cancer cells (Fosslien, 2008; Oronsky et al., 2014; Poljsak et al., 2019; Szent-Gyorgyi, 1977; Seyfried, 2015; Seyfried et al., 2019). Indeed, unbridled proliferation was the dominant growth phenotype of all ancient organisms before oxygen entered the atmosphere about 2.5 billion years ago (Poljsak et al., 2019; Szent-Gyorgyi, 1977). The dependency of tumor cells on glycolysis and glutaminolysis will also make them resistant to apoptosis and chemotherapeutic drugs (Xu et al., 2005). The activity of the p-glycoprotein, which protects cells from toxic chemotherapy, is driven by glycolysis (Horio et al., 1988; Xu et al., 2005). The rewiring of ATP synthesis from OxPhos to fermentation involving SLP would cause a cell to enter its default state with consequent dedifferentiation, apoptotic resistance, and unbridled proliferation, i.e., neoplasia (Seyfried et al., 2014; Szent-Gyorgyi, 1977; Soto and Sonnenschein, 2004).

**Figure 5:**
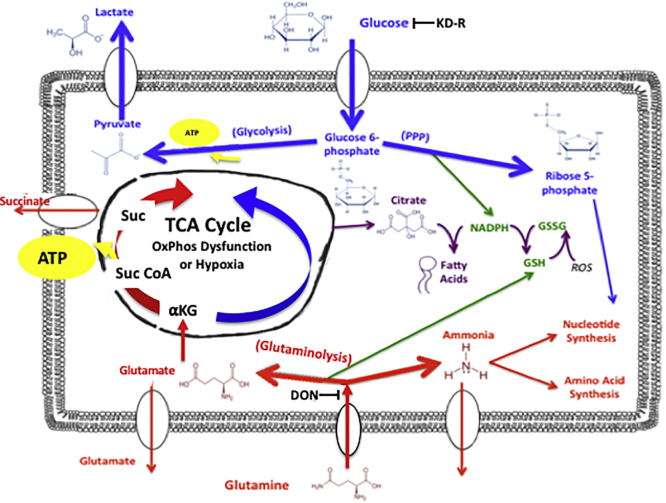
Glucose and Glutamine Targeting for the Metabolic Management of Cancer Most if not all tumor cells, regardless of tissue origin, are largely dependent on glucose and glutamine for ATP synthesis, growth,and survival (Mukherjee et al., 2019; Seyfried et al., 2020). ATP synthesis through SLP in the cell cytoplasm (glycolysis) and in the TCA cycle (glutaminolysis) will compensate for diminished ATP synthesis arising from defective OxPhos or hypoxia that occurs in many tumors. The calorie-restricted ketogenic diet (KD-R) will reduce glucose carbons for both the glycolytic pathway and the pentose phosphate pathway (PPP) that supply ATP and metabolic precursors for synthesis of glutathione, lipids, and nucleotides. Glutaminase inhibitors, like 6-diazo-5-oxo-L-norleucine (DON), will deplete glutamate and the glutamine-derived amide nitrogen needed for ammonia and nucleotide synthesis (Nilsson et al., 2020). Depletion of glutamine-derived glutamate will reduce anapleurotic carbons to the TCA cycle through *α*-KG for protein synthesis, while also reducing energy production through the succinyl-CoA ligase reaction in the TCA cycle. Glutamine-derived glutamate is also used for glutathione production, which protects tumor cells from oxidative stress. The KD-R + DON will make tumor cells vulnerable to lethal oxidative stress. The simultaneous targeting of glucose and glutamine, using the KD-R + DON, will thus starve tumor cells of ATP synthesis while also blocking their ability to synthesize lipids, proteins, and nucleotides. Metabolic starvation could also reduce extracellular acidification through reduction of lactate and succinate, the end products of glucose and glutamine fermentation, respectively. The elevation of non-fermentable ketone bodies is tumor cell toxic and is unable to replace glucose and glutamine for energy. Importantly, ketone bodies protect normal cells from oxidative stress while providing an alternative energy source to glucose. Figure and legend modified with permission from (Seyfried et al., 2017; Mukherjee et al., 2019).

Efforts to target glucose and glutamine simultaneously show promise as a therapeutic strategy for managing a broad range of cancers (Cervantes-Madrid et al., 2015; Mukherjee et al., 2019; Leone et al., 2019; Reckzeh et al., 2019; Oronsky et al., 2014; Mendez-Lucas et al., 2020). Leone et al. showed that an analog of the pan glutaminase inhibitor, 6-diazo-5-oxo-L-norleucine (DON), not only inhibited glutamine metabolism but also inhibited glycolysis and related pathways, thus disabling the Warburg effect and significantly reducing tumor growth (Leone et al., 2019). Similar observations in reducing tumor growth were observed in combining glycolysis inhibitors (Glutor or lonidamine) with either DON or the glutaminase inhibitor CB-839 (Cervantes-Madrid et al., 2015; Reckzeh et al., 2019). We also found a powerful therapeutic synergy in combining DON with a calorie-restricted ketogenic diet (KD-R) for managing late-stage growth in the VM-M3 and CT-2A syngeneic glioblastoma mouse tumors (Mukherjee et al., 2019). The KD-R not only reduced the ratio of glucose to non-fermentable ketone bodies in the blood but also facilitated delivery of DON to the tumors through the blood-brain barrier. This therapeutic strategy reduced simultaneously the availability of glucose and glutamine to the glycolytic and glutaminolysis pathways in the tumor cells. Moreover, ketone bodies enhance the metabolic efficiency of normal host cells, but are growth inhibitory or even toxic to many tumor cells (Clarke et al., 2012; Veech, 2004; Cahill and Veech, 2003; Bartmann et al., 2018; Skinner et al., 2009; Fine et al., 2009; Poff et al., 2017; Magee et al., 1979; Hagihara et al., 2020; Ji et al., 2020). Abnormalities in cardiolipin and other phospholipids in the inner mitochondrial membranes would prevent tumor cells from using ketone bodies for ATP synthesis (El Kebbaj et al., 1986; Kiebish et al., 2008, 2009). Through their anti-angiogenic, anti-inflammatory, and pro-apoptotic actions, calorie restriction and KD-R can normalize the tumor microenvironment (Zhou et al., 2007; Marsh et al., 2008; Mukherjee et al., 2002, 2004, 2019; Mulrooney et al., 2011; Shelton et al., 2010a; Urits et al., 2012). Maximal therapeutic benefit of glutamine-targeting drugs can be obtained for a broad range of cancers when administered to patients under reduced blood glucose and elevated ketone bodies, i.e., nutritional ketosis (Seyfried et al., 2017; Weber et al., 2019; Klement, 2017; Winter et al., 2017; Poff et al., 2017). Figure 5 summarizes how the simultaneous targeting of glucose and glutamine could help manage tumor growth. The simultaneous targeting of glucose and glutamine, while under nutritional ketosis, will selectively disrupt ATP synthesis in OxPhos-deficient cancer cells thus leading to catastrophic energy failure and their death.

## Limitations

There are a number of issues that must also be addressed regarding our position to provide an objective and balanced interpretation of the data supporting the presented information. We recognize that the data presented in our review can have additional interpretations and limitations. There is now substantial evidence showing that glucose and glutamine are major fuels for cancer growth and that multiple oncogenes enable the metabolic rewiring from OxPhos to fermentation metabolism in a broad range of cancers (Still and Yuneva, 2017; Yuneva, 2008; Tardito et al., 2015; Zielinski et al., 2017). Besides evidence from numerous *in vitro* studies, *in vivo* studies have also recognized the importance of glucose and glutamine in driving tumor growth (Mendez-Lucas et al., 2020; Leone et al., 2019; Mukherjee et al., 2019; Cervantes-Madrid et al., 2015). Labeled glucose and glutamine analogs are used clinically to monitor patient tumor tissues for purposes of diagnosis and therapy (Vander Heiden et al., 2009; Baguet et al., 2020; Qu et al., 2012). Although glucose-dependent lactic acid fermentation is seen in the majority of cancers, regardless of tumor cell type, stage of growth, or genotype (Yu et al., 2019; Vander Heiden et al., 2009), glutamine utilization seems to be associated more with tumors of mesenchymal phenotype than for tumors with stem cell phenotype (Mukherjee et al., 2019; Oizel et al., 2017). The cellular mesenchymal phenotype has been linked to findings that many metastatic cancers express characteristics of macrophages, which are of mesenchymal origin (Garvin et al., 2019; Clawson et al., 2015; Pawelek, 2008; Seyfried and Huysentruyt, 2013; Ruff and Pert, 1984). Glutamine is a major fuel for cells of the immune system, especially macrophages (Piva et al., 1991; Newsholme, 2001), and is a driver of metastasis (Oh et al., 2020; Shelton et al., 2010b; Taus et al., 2017). Ketone bodies can spare muscle protein and thus delay cancer cachexia because metastatic cancer cells are major consumers of muscle-derived glutamine (Koutnik et al., 2020; Tisdale and Brennan, 1988; Tisdale et al., 1987). As hypoxia also contributes to metastasis, anti-angiogenic drugs like bevacizumab would be expected to increase tumor invasion and distant metastasis, and should be avoided (Donato et al., 2020; Seyfried et al., 2019). The recent genomic data from Yuan et al. have identified numerous pathological mutations in mtDNA thus linking gene mutations to abnormal mitochondrial function, especially involving OxPhos (Yuan et al., 2020). The role of genetic heterogeneity and whole-genome-dependency data on the origin and progression of cancer have been addressed in other reviews (Martincorena and Campbell, 2015; Martincorena et al., 2018; Yokoyama et al., 2019; Yizhak et al., 2019; Seyfried, 2015; Baker, 2015; Soto and Sonnenschein, 2004). We recognize that the information presented supporting our position can have additional interpretations. Further studies will be necessary to provide a more complete and realistic interpretation of how the genomic abnormalities can be integrated with the recognized abnormalities in bioenergetics/metabolism in cancer.

## General Conclusions

Information is reviewed showing how tumor cells can synthesize ATP when OxPhos is reduced or absent. Glutamine-driven mSLP can compensate in part for insufficient ATP synthesis through OxPhos in cancer cells, most of which have documented anomalies in the number, structure, and function of mitochondria. Glutamine-generated ATP synthesis through mSLP can also compensate for reduced ATP synthesis from glycolysis in tumor cells that express the PKM2 isoform. The protracted loss of ATP synthesis through OxPhos coupled with compensatory increases in cytoplasmic and mitochondrial SLP underlies the transition from regulated growth to dysregulated growth, i.e., neoplasia. Oncogene activation is necessary to enable SLP through the glycolysis and glutaminolysis pathways. Recognition of mSLP as alternative energy mechanism to OxPhos can help resolve misinformation on oxygen consumption, fatty acid oxidation, and controversies associated with the origin of ATP synthesis in cancer. As glucose and glutamine are the prime fuels for driving tumor growth, therapeutic strategies that can simultaneously target the availability of these fuels should have potential in improving progression-free and overall survival for most patients with cancer.
